# Prediction of lymphoma response to CAR T cells by deep learning-based image analysis

**DOI:** 10.1371/journal.pone.0282573

**Published:** 2023-07-21

**Authors:** Yubing Tong, Jayaram K. Udupa, Emeline Chong, Nicole Winchell, Changjian Sun, Yongning Zou, Stephen J. Schuster, Drew A. Torigian

**Affiliations:** 1 Medical Image Processing Group, Department of Radiology, University of Pennsylvania, Philadelphia, Pennsylvania, United States of America; 2 Lymphoma Program, Abramson Cancer Center, Perelman Center for Advanced Medicine, University of Pennsylvania, Philadelphia, Pennsylvania, United States of America; City of Hope, UNITED STATES

## Abstract

Clinical prognostic scoring systems have limited utility for predicting treatment outcomes in lymphomas. We therefore tested the feasibility of a deep-learning (DL)-based image analysis methodology on pre-treatment diagnostic computed tomography (dCT), low-dose CT (lCT), and 18F-fluorodeoxyglucose positron emission tomography (FDG-PET) images and rule-based reasoning to predict treatment response to chimeric antigen receptor (CAR) T-cell therapy in B-cell lymphomas. Pre-treatment images of 770 lymph node lesions from 39 adult patients with B-cell lymphomas treated with CD19-directed CAR T-cells were analyzed. Transfer learning using a pre-trained neural network model, then retrained for a specific task, was used to predict lesion-level treatment responses from separate dCT, lCT, and FDG-PET images. Patient-level response analysis was performed by applying rule-based reasoning to lesion-level prediction results. Patient-level response prediction was also compared to prediction based on the international prognostic index (IPI) for diffuse large B-cell lymphoma. The average accuracy of lesion-level response prediction based on single whole dCT slice-based input was 0.82+0.05 with sensitivity 0.87+0.07, specificity 0.77+0.12, and AUC 0.91+0.03. Patient-level response prediction from dCT, using the “Majority 60%” rule, had accuracy 0.81, sensitivity 0.75, and specificity 0.88 using 12-month post-treatment patient response as the reference standard and outperformed response prediction based on IPI risk factors (accuracy 0.54, sensitivity 0.38, and specificity 0.61 (p = 0.046)). Prediction of treatment outcome in B-cell lymphomas from pre-treatment medical images using DL-based image analysis and rule-based reasoning is feasible. This approach can potentially provide clinically useful prognostic information for decision-making in advance of initiating CAR T-cell therapy.

## Introduction

Autologous CD19-directed chimeric antigen receptor (CAR) modified T-cell therapy has improved the prognosis for adult patients with relapsed or refractory aggressive B-cell lymphomas [1–5]. Research to identify biomarkers of response to CAR T-cell therapies has focused on laboratory and/or pathology-based analyses. However, a radiologic image-based approach to determine personalized prediction of response to CAR T-cell therapies would have unique advantages including use of existing diagnostic images previously acquired for clinical purposes, lack of invasiveness, availability of information regarding the regional properties of disease sites and unaffected organs body-wide, and productive efficiency.

Currently, deep learning (DL) techniques show considerable promise in image classification [6–9], segmentation, and pattern recognition [10–15], and outperform most traditional machine learning approaches due to their uncanny ability to learn local image patterns that far exceed the ability of classical and handcrafted methods. Transfer learning is a DL approach in which a trained model (DL network) for one task is used as a starting point to continue to train the model for another task. It can improve the prediction accuracy for DL neural networks that were trained with only medical images [16, 17]. For example, AlexNet is a common neural network used in transfer learning which has been trained on millions of non-medical images and widely adopted to classify images [18].

The purpose of this study is to assess the feasibility of a DL-based image analysis methodology applied to pre-treatment diagnostic computed tomography (dCT) images, low-dose CT (lCT) images from positron emission tomography/computed tomography (PET/CT) scans, and 18F- fluorodeoxyglucose (FDG) PET images from PET/CT scans to predict lesion-level treatment response to CAR T-cell therapy, and to apply a rule-based reasoning methodology to DL output to predict patient-level response for patients with diffuse large B-cell lymphoma (DLBCL) and follicular lymphoma (FL).

## Materials and methods

This retrospective study was conducted following approval from the Institutional Review Board at the University of Pennsylvania along with a Health Insurance Portability and Accountability Act waiver.

### Study cohort and data sets

**Fig 1:**
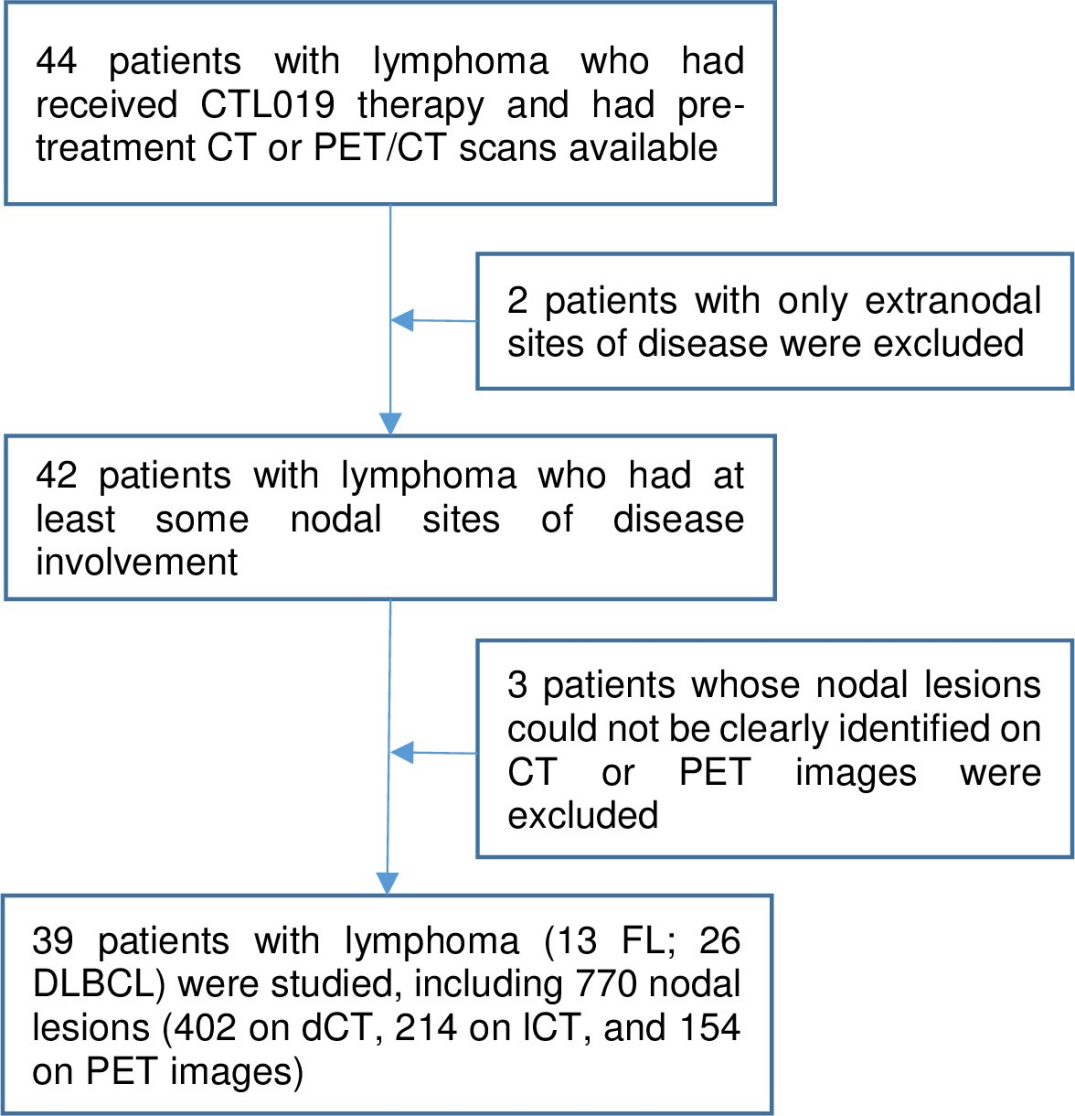
Patient inclusion and exclusion schema for this study.

Pre-treatment diagnostic CT and PET/CT images of the neck, chest, abdomen, and pelvis previously obtained on a clinical research protocol using autologous T cells that express a CD19-directed CAR (CTL019, later designated tisagenlecleucel) to treat patients with relapsed or refractory DLBCL or FL (ClinicalTrials.gov number, NCT02030834) were utilized for this study. This study included response prediction at both the lesion level and the patient level, which included 26 patients (20M, 6F; median age 57 years (range 28–74)) with DLBCL and 13 patients (7M, 6F; median age 62 years (range 43–72)) with FL. The patient inclusion and exclusion schema are shown in **Fig 1**.

All individual lymph node disease sites were identified using pre-treatment images followed by determination of ground truth lesion-level responses for all individual nodal lesions via comparison of pre-treatment and post-treatment images by an expert radiologist. Lesion-level response to treatment was defined by interval decrease in size or metabolic activity or interval resolution of a lesion between pre-treatment and post-treatment images, whereas lesion-level non-response was defined as lack of change or interval increase in size or metabolic activity of a lesion. Post-treatment dCT and PET/CT images utilized to determine ground truth lesion-level responses were acquired 94.0±33.2 days after pre-treatment images. Extranodal lesion sites, as well as splenic and Waldeyer’s ring nodal-equivalent lesions, were not considered given the small number of lesions encountered at these anatomic locations. The number of International Prognostic Index (IPI) risk factors present at the time of pre-treatment imaging for each patient with DLBCL was recorded [19]; 8 patients had 0–1 risk factors (low risk group), 11 patients had 2 (low-intermediate-risk group), 4 patients had 3 (high-intermediate risk group), and 3 patients had 4–5 (high risk group). Patient-level response status based on post-treatment scans acquired 12 months after pre-treatment scans was also determined for all patients.

### DL for lesion-level treatment response prediction and evaluation

Lesion-level response prediction was performed by using volume of interest (VOI)-based and whole slice-based (non-VOI) approaches using CAVASS software [20] to place a rectangular box around each abnormal lymph node in 3D space (see supplemental text for details regarding VOI settings). Every lesion was labeled with a 0 or 1, where 0 indicated a lesion without response to treatment and 1 indicated a lesion with response to treatment. In total, 770 lymph node lesions (402 by dCT; 214 by lCT; 154 by PET) were assessed (see supplemental **S1 Table** for response category details). Five input scenarios were considered for the DL network: A single VOI-restricted image slice passing through the mid-portions of lesions (1 VOI-slice), three contiguous VOI-restricted image slices passing through the mid-portions of lesions (3 VOI-slices), a single whole-image slice passing through the mid-portions of lesions (1 whole-slice), three contiguous whole-image slices passing through the mid-portions of lesions (3 whole-slices), and combined single VOI-restricted and single whole-image slices passing through the mid-portions of lesions in two channels of one input sample (combined-slices). Axial slices were selected so as to avoid having different lesions within the same whole slice. In total, 15 combinations (5 input scenarios × 3 image modalities) were tested. To improve test statistics, multi-fold cross validation was conducted by repeating each experiment 10 times for different combinations of 6:2:2 (training: validation: testing) data set division. Data augmentation [21, 22] was used on training data sets to improve training performance. In total, 3040 experiments were conducted (2400 with transfer learning and 640 with incremental learning). Transfer learning was performed by loading a pre-trained neural network (AlexNet [18]), modifying its output layers/decision by replacing the last three layers with a fully connected layer, a Softmax layer, and a binary classification output layer for the specific classification purpose, and retraining the network with specific training samples. AlexNet has a simple structure (with only 5 convolutional layers) and is more easily retrained to test the proposed approach. Although only the pre-trained “AlexNet” was used here, the same framework can be easily configured using other more recent pre-trained neural networks such as VGG [23] or ResNet [24] (with SGD [25]). Incremental learning was performed to predict response on lCT and PET by employing a dCT model utilized in transfer learning, and then finely tuning this model by using lCT and PET training samples. Please see the supplemental text, **S2 Table** and **S1** and **S2 Figs** for further details regarding data augmentation and the deep learning experimental set up.

The accuracy, sensitivity, and specificity of the lesion-level prediction task and the area under the curve (AUC) for the receiver operating characteristic (ROC) curve were then evaluated. Two-sided t-testing was utilized to compare experimental results from different input scenarios, hyperparameter settings, and image modalities. A p value of < 0.05 was considered as statistically significant.

### Rule-based reasoning for patient-level treatment response prediction and evaluation

Patient-level response prediction was subsequently performed in all 39 patients using a rule-based reasoning approach applied to lesion-level prediction results from the DL network. After lesion-level response was predicted using transfer learning, two rules, the “All” rule and the “Majority” rule, were utilized to determine patient-level response. For the “All” rule, a patient responder is one in whom all lesions have responded, and a patient non-responder is one in whom at least one lesion has not responded. For the “Majority” rule, a patient responder is one in whom the majority of all lesions have responded (using thresholds of either 60% for the “Majority 60%” rule or 70% for the “Majority 70%” rule), and a patient non-responder is one in whom the majority of lesions (using thresholds of either 60% or 70% of all lesions) have not responded. The reference standard for patient-level response (responder/non-responder) was based on the findings on cross-sectional imaging scans acquired 12-months after the date of pre-treatment scans. Also, since the IPI is currently used in clinical practice to assess risk in patients with DLBCL, we compared its performance to that of our rule-based method in the 26 patients with DLBCL (10 responders and 16 non-responders). For the IPI method, DLBCL patients were categorized into responder and non-responder groups by using different thresholds based on the number of IPI risk factors (IPI ≤1, IPI ≤2, and IPI ≤3), where the lower number groups were considered as responder groups. The accuracy, sensitivity, and specificity of the patient-level prediction task were then evaluated for rule-based and IPI-based approaches, utilizing Pearson’s chi-square test for statistical comparisons.

## Results

### Lesion-level treatment response prediction results

**Fig 2:**
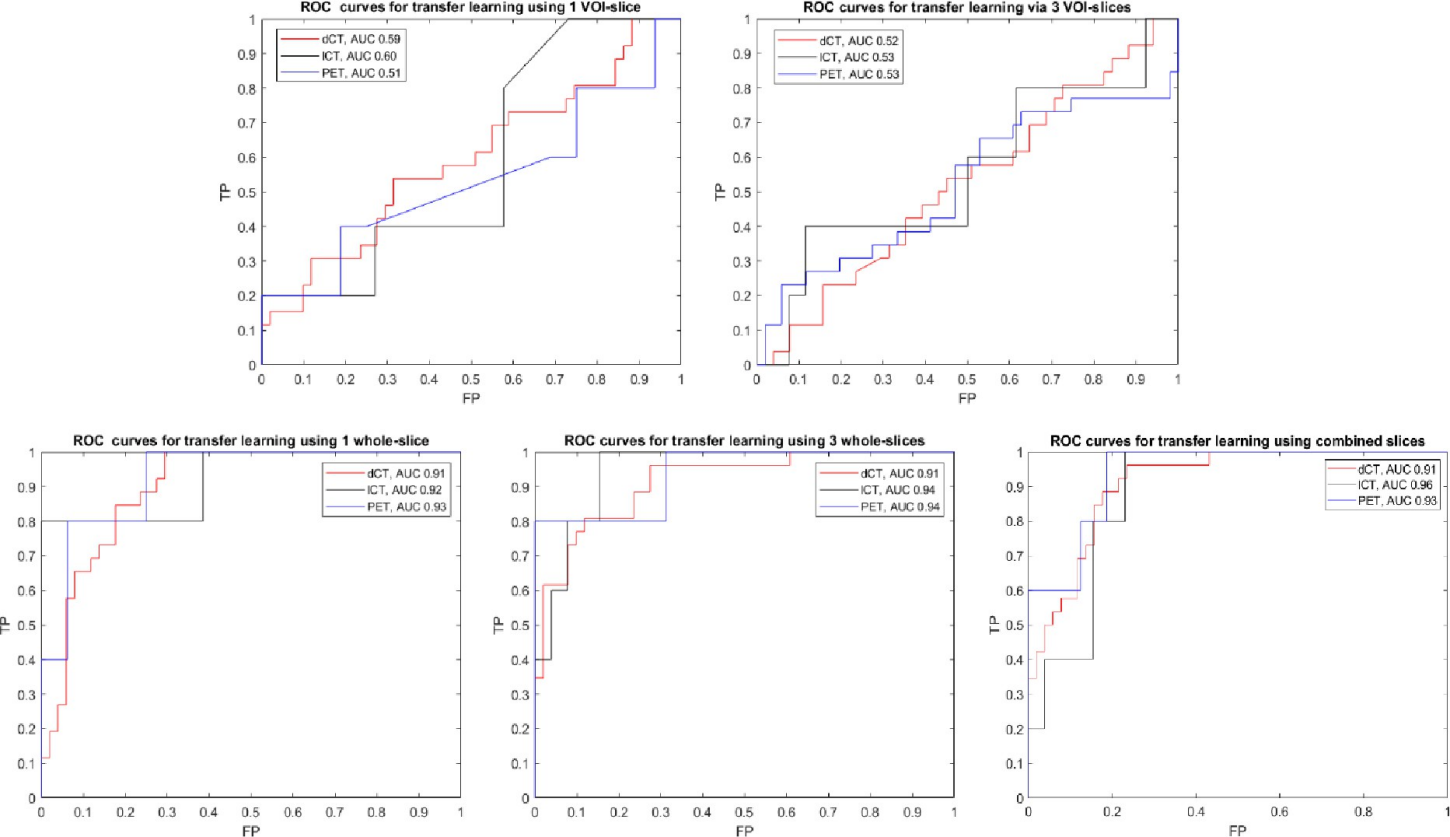
Receiver operator characteristic (ROC) curves for diagnostic performance of lesion-level treatment response prediction in lymphoma using transfer learning (on test data sets for 5 input scenarios and 3 image modalities using 40 epochs and batch size 5). TP = true positive fraction, FP = false positive fraction, AUC = area under the curve, dCT = diagnostic computed tomography, lCT = low-dose computed tomography, PET = positron emission tomography.

The diagnostic performance results of lesion-level response prediction using transfer learning for the five input scenarios from dCT, lCT, and PET image modalities are shown in **Table 1** (with p values provided in **S3** and **S4** Tables), and the ROC curves are shown in **Fig 2**. The predictive performances of 1 VOI-slice and 3 VOI-slices input scenarios on dCT, lCT, and PET were substantially lower than those of the corresponding whole slice-based input scenarios. For example, the accuracy of 1 VOI-slice vs. 1 whole-slice input from dCT was 0.68±0.05 vs. 0.82±0.05, respectively (p < 0.0001) with AUC 0.59±0.04 vs. 0.91±0.03, respectively (p < 0.0001), and the accuracy of 3 VOI-slices vs. 3 whole-slices input from dCT was 0.65±0.05 vs. 0.84±0.05, respectively (p < 0.0001) with AUC 0.52±0.07 vs. 0.90±0.05, respectively (p < 0.0001). The predictive performances of 1 whole-slice and 3 whole-slices inputs from dCT (AUC 0.91±0.03 vs. 0.90±0.05, respectively, p = 0.435) were similar, as were those from lCT (AUC 0.92±0.08 vs. 0.94±0.07, respectively, p = 0.66) and from PET (AUC 0.93±0.07 vs. 0.95±0.06, respectively, p = 0.46). The predictive performances of combined-slices input from dCT, lCT, or PET were not statistically different from those based on 1 whole-slice input. For dCT, lesion-level response prediction using 1 whole-slice input had accuracy 0.82+0.05, sensitivity 0.87+0.07, specificity 0.77+0.12, and AUC 0.91+0.03. For lCT, lesion-level response prediction using 1 whole-slice input had accuracy 0.91+0.06, sensitivity 0.94+0.06, specificity 0.75+0.32, and AUC 0.92+0.08.

**Table 1:** Diagnostic performance of lesion-level treatment response prediction in lymphoma using transfer learning (for 5 input scenarios and 3 image modalities using 40 epochs and batch size 5). Mean and standard deviation values are displayed. VOI = volume of interest, dCT = diagnostic computed tomography, lCT = low-dose computed tomography, PET = positron emission tomography, Acc = accuracy, Sens = sensitivity, Spec = specificity, AUC = area under the curve.

	dCT				lCT				PET			
Input scenario	Acc	Sens	Spec	AUC	Acc	Sens	Spec	AUC	Acc	Sens	Spec	AUC
1 VOI-slice	0.68 ±0.05	0.70 ±0.02	0.58 ±0.16	0.59 ±0.04	0.68 ±0.05	0.70 ±0.02	0.58 ±0.16	0.60 ±0.11	0.68 ±0.05	0.70 ±0.02	0.58 ±0.16	0.51 ±0.14
1 whole-slice	0.82 ±0.05	0.87 ±0.07	0.77 ±0.12	0.91 ±0.03	0.91 ±0.06	0.94 ±0.06	0.75 ±0.32	0.92 ±0.08	0.87 ±0.06	0.90 ±0.06	0.77 ±0.19	0.93 ±0.07
3 VOI-slices	0.65 ±0.05	0.68 ±0.02	0.52 ±0.21	0.52 ±0.07	0.79 ±0.06	0.84 ±0.02	0.21 ±0.33	0.53 ±0.17	0.65 ±0.02	0.68 ±0.01	0.48 ±0.09	0.53 ±0.07
3 whole-slices	0.84 ±0.05	0.90 ±0.04	0.76 ±0.12	0.90 ±0.05	0.90 ±0.05	0.95 ±0.04	0.74 ±0.20	0.94 ±0.07	0.90 ±0.07	0.95 ±0.05	0.81 ±0.19	0.95 ±0.06
combined-slices	0.84 ±0.04	0.89 ±0.03	0.76 ±0.10	0.91 ±0.03	0.93 ±0.03	0.96 ±0.03	0.83± 0.14	0.98 ±0.02	0.87 ±0.07	0.93 ±0.07	0.73 ±0.16	0.92 ±0.08

For PET, 1 whole-slice input had accuracy 0.87+0.06, sensitivity 0.90+0.06, specificity 0.77+0.19, and AUC 0.93+0.07. Although the accuracy of 1 whole-slice input from dCT was lower than the accuracy from lCT (p = 0.002) and PET (p = 0.08), the AUC and specificity of 1 whole-slice input did not statistically differ between dCT, lCT, and PET. There were no significant differences in lesion-level response prediction accuracy or AUC between transfer learning and incremental learning approaches using 1 whole-slice input from lCT or PET. Further details regarding experimental set up and results for lesion-level response prediction based on transfer learning, incremental learning, different input scenarios, different image modalities, and different hyperparameter settings are included in the supplemental text, **S5–S8 Tables** and **S3 and S4 Figs**.

### Patient-level treatment response prediction results

The results of patient-level response prediction using the rule-based reasoning approach from dCT, lCT, and PET relative to the reference standard of 12-month post-treatment patient-level response status are shown in **Table 2**. These were derived from lesion-level response predictions based on the 1 whole-slice input scenario and transfer learning. (Comparable results based on the 3 whole-slices input scenario are reported separately in supplemental **S9 Table**). Patient-level response prediction for all patients from dCT based on the “Majority 60%” rule had accuracy 0.79, sensitivity 0.83, and specificity 0.75, which was not significantly different than that from lCT (with accuracy 0.65, sensitivity 0.60, and specificity 0.75) (p = 0.80) and PET (with accuracy 0.56, sensitivity 0.55, and specificity 0.57) (p = 0.87). In addition, patient-level response prediction for DLBCL patients from dCT based on the “Majority 60%” rule had accuracy 0.81, sensitivity 0.75, and specificity 0.88, which was significantly better than the best IPI-based patient-level response prediction using an IPI risk factor threshold of <1 (with accuracy 0.54, sensitivity 0.38, and specificity 0.61) (p = 0.046).

**Table 2:** Diagnostic performance of patient-level treatment response prediction in lymphoma using rule-based reasoning approach (from lesion-level response predictions using 1 whole-slice input scenario, 3 image modalities, and transfer learning) compared to International Prognostic Index risk factors for diffuse large B-cell lymphoma (DLBCL) patients. Note that results are shown for entire subject cohort (All) and for DLBCL subject cohort. dCT = diagnostic computed tomography, lCT = low-dose computed tomography, PET = positron emission tomography, IPI = International Prognostic Index, Acc = accuracy, Sens = sensitivity, Spec = specificity.

		Patient response with "All" Rule			Patient response with "Majority" Rule					
Subject cohort	Modality	All lesions Responded			At least "60%" lesions responded			At least "70%" lesions responded		
		Acc	Sens	Spec	Acc	Sens	Spec	Acc	Sens	Spec
**All**	**dCT**	0.61	1.00	0.56	0.79	0.83	0.75	0.71	0.88	0.65
	**lCT**	0.52	0.50	0.54	0.65	0.60	0.75	0.65	0.60	0.75
	**PET**	0.61	0.60	0.63	0.56	0.55	0.57	0.61	0.58	0.67
**DLBCL**	**dCT**	0.69	1.00	0.64	0.81	0.75	0.88	0.75	0.80	0.73
	**lCT**	0.56	0.44	0.67	0.56	0.45	0.71	0.56	0.45	0.71
	**PET**	0.57	0.50	0.67	0.43	0.38	0.50	0.50	0.44	0.60
	**IPI ≤ 1**	Acc = 0.54; Sens = 0.38; Spec = 0.61								
	**IPI ≤ 2**	Acc = 0.42; Sens = 0.37; Spec = 0.57								
	**IPI ≤ 3**	Acc = 0.27; Sens = 0.30; Spec = 0.00								

For dCT, the accuracy of the “Majority 60%” rule (0.79) was not statistically significantly different than that of the “Majority 70%” rule (0.71) (p = 0.38) but was statistically significantly greater than that of the “All” rule (0.61) (p = 0.027). For lCT, the accuracies of the “Majority 60%” and “Majority 70%” rules (0.65) were identical and not statistically significantly different than that of the “All” rule (0.52) (p = 0.20). For PET, the accuracies of the “Majority 70%” and “All” rules (0.61) were identical and not statistically significantly different than that of the “Majority 60%” rule (0.56) (p = 0.73).

## Discussion and conclusions

In this study, we investigated the feasibility of a novel deep learning image analysis methodology applied to pre-treatment diagnostic CT, low-dose CT, and FDG-PET images to predict lesion-level treatment response to CAR T-cell therapy in patients with lymphoma, and then used a rule-based reasoning approach to assess the feasibility of predicting patient-level response. To our knowledge, such approaches have not yet been studied in this clinical context.

We showed that prediction of treatment outcome in B-cell lymphomas from pre-treatment medical images using DL-based image analysis at the lesion level and rule-based reasoning at the patient level is feasible at a high level of accuracy. We also demonstrated that patient-level response prediction using rule-based reasoning outperformed prediction based on clinical IPI risk factors in patients with DLBCL.

Recent research on outcome prediction in patients with DLBCL using regression and machine learning methods has focused on use of clinical and pathologic information. For example, Galaznik *et al* created a model for predicting health outcome in patients with DLBCL treated with standard of care by using lasso logistic regression [26]. Biccler *et al* used a machine learning approach to achieve optimum outcome prediction in patients with DLBCL, which combined several predicted survival curves into one by means of a weighted average [27]. The weights were selected so that the cross-validated integrated Brier score (IBS) was minimized, and different models, such as Cox proportional hazard (CPH) model, penalized CPH models, and accelerated failure time (AFT) model, were selected for forming survival curves. Biccler *et al* reported a concordance index (an AUC) from C-Statistic of 0.756 for Danish and 0.744 for Swedish cohorts [26, 27]. Reinart *et al* reported on the value of CT-based textural features and volume-based PET parameters for response assessment in patients with DLBCL undergoing CAR T-cell therapy [28]. Although they showed that certain tumor features at baseline such as whole-body metabolic tumor volume, whole-body total lesion glycolysis, and CT-based texture properties were statistically significantly different between patients with complete response vs. those with partial response to treatment, no prediction analysis was actually performed based on baseline imaging features and no separate testing data set was utilized.

Although deep learning based prediction typically requires a large amount of training samples, once the training procedure has been completed, the approach is fully automatic. Furthermore, it can be performed in an end-to-end mode without need for hand-crafted features since optimal features are automatically extracted and refined during training, in contradistinction to traditional image analysis approaches. Also, deep learning has a good non-linear regression ability and can handle multiple high dimensional and complex features, which may be challenging for traditional image analysis methods. Use of an image-based approach to predict tumor treatment response has advantages compared to a pathology-based approach, given that pathology information may not always be available at baseline and requires an invasive procedure to obtain tumor samples, is reflective only of the properties of those specific tumor lesions that were sampled which may or may not be representative of other tumor lesions in the body, and does not provide information about the quantity or spatial distribution of tumor throughout the body.

One limitation of this study is the relatively small number of patients who were assessed, which precluded use of machine learning approaches for patient-level response prediction. However, a large number of individual lymphoma lesions were available for evaluation in these patients and data augmentation techniques were utilized, enabling high diagnostic performance of lesion-level response prediction. Furthermore, we were still able to achieve a high diagnostic performance of patient-level prediction using a rule-based reasoning approach. One other limitation is that we restricted our attention to lymph node lesions only, given the small numbers of extranodal, splenic, and Waldeyer’s ring lesions in our patient cohort. We may include other such lesion inputs in future larger scale studies.

In summary, we have demonstrated the feasibility of a novel deep learning image analysis methodology using pre-treatment CT and PET/CT images to accurately predict lesion-level responses in patients with lymphomas treated with CAR T-cell therapy. We also demonstrate the feasibility of using a rule-based reasoning approach to accurately predict patient outcomes. Our results suggest that these approaches may provide new information that can be used to predict which patients will or will not respond to treatment in advance of initiating therapy.

## Supporting information

S1 Fig Strategy of transfer learning and incremental learning utilized for lesion-level response prediction. (TIF)

S2 Fig Deep learning-based architecture utilized for lesion-level treatment response prediction. CNN = convolutional neural network, ReLU = rectified linear unit, Conv. = convolutional.
(TIF)

S3 Fig Receiver operator characteristic (ROC) curves for diagnostic performance of lesion-level treatment response prediction in lymphoma using incremental learning vs. transfer learning (on test data sets for 1 whole-slice and 3 whole-slice input scenarios and 3 image modalities using 40 epochs and batch size 5). TP = true positive fraction, FP = false positive fraction, AUC = area under the curve, dCT = diagnostic computed tomography, lCT = low-dose computed tomography, PET = positron emission tomography.
(TIF)

S4 Fig Training / validation curves from one of the 10 repeat experiments on diagnostic computed tomography (dCT) using transfer learning with batch size (B) = 5 and number of epochs (E) = 80. (TIF)

S1 Table Summary of response categories of lymphoma patients who received CAR T-cell therapy. Patients categorized as (1) full responders (F-R) (i.e., where all lesions responded), (2) full non-responders (F-NR) (i.e., where no lesions responded), and (3) partial responders (P-R) (i.e., where only some lesions responded). dCT = diagnostic computed tomography, lCT = low-dose computed tomography, PET = positron emission tomography.
(DOCX)

S2 Table Experiments with transfer learning for lesion-level treatment response prediction. dCT = diagnostic computed tomography, lCT = low-dose computed tomography, PET = positron emission tomography, VOI = volume of interest.
(DOCX)

S3 Table P values of t-test comparisons of diagnostic performance between 5 input scenarios for lesion-level treatment response prediction in lymphoma. Cells with statistically significant p values are highlighted. dCT = diagnostic computed tomography, lCT = low-dose computed tomography, PET = positron emission tomography, VOI = volume of interest, Acc = accuracy, Sens = sensitivity, Spec = specificity, AUC = area under the curve.
(DOCX)

S4 Table P values of t-test comparisons of diagnostic performance between 3 image modalities (for 1 whole-slice and 3 whole-slices input scenarios) for lesion-level treatment response prediction. Cells with statistically significant p values are highlighted. dCT = diagnostic computed tomography, lCT = low-dose computed tomography, PET = positron emission tomography, Acc = accuracy, Sens = sensitivity, Spec = specificity, AUC = area under the curve.
(DOCX)

S5 Table **a.** Diagnostic performance of lesion-level treatment response prediction in lymphoma from diagnostic computed tomography (dCT) images for 5 input scenarios (using 40 epochs and batch size 5). Mean and standard deviation values are displayed. VOI = volume of interest, AUC = area under the curve. **b.** Diagnostic performance of lesion-level treatment response prediction in lymphoma from low-dose computed tomography (lCT) images for 5 input scenarios (using 40 epochs and batch size 5). Mean and standard deviation values are displayed. VOI = volume of interest, AUC = area under the curve. **c.** Diagnostic performance of lesion-level treatment response prediction in lymphoma from positron emission tomography (PET) images for 5 input scenarios (using 40 epochs and batch size 5). Mean and standard deviation values are displayed. VOI = volume of interest, AUC = area under the curve.
(ZIP)

S6 Table Diagnostic performance of lesion-level treatment response prediction in lymphoma using incremental learning vs. transfer learning (for 2 input scenarios) on low-dose computed tomography (lCT) and positron emission tomography (PET) image modalities. Mean and standard deviation values are displayed. Acc = accuracy, Sens = sensitivity, Spec = specificity, AUC = area under the curve.
(DOCX)

S7 Table Diagnostic performance of lesion-level treatment response prediction in lymphoma using transfer learning on 1 whole-slice input scenario from diagnostic computed tomography (dCT) based on different hyperparameters of batch size (B) and number of epochs (E). Mean and standard deviation values are displayed. Acc = accuracy, Sens = sensitivity, Spec = specificity, AUC = area under the curve.
(DOCX)

S8 Table P values of t-test comparisons of diagnostic performance between selected hyperparameter combinations of transfer learning (from S7 Table) for lesion-level treatment response prediction (using 1 whole-slice input scenario from diagnostic computed tomography (dCT)). Cells with statistically significant p values are highlighted. B = batch size, E = number of epochs, Acc = accuracy, Sens = sensitivity, Spec = specificity, AUC = area under the curve.
(DOCX)

S9 Table Diagnostic performance of patient-level treatment response prediction in lymphoma using rule-based reasoning approach (from lesion-level response predictions using 3 whole-slices input scenario, 3 image modalities, and transfer learning) compared to International Prognostic Index risk factors for diffuse large B-cell lymphoma (DLBCL) patients. Note that results are shown for entire subject cohort (All) and for DLBCL subject cohort. dCT = diagnostic computed tomography, lCT = low-dose computed tomography, PET = positron emission tomography, IPI = International Prognostic Index, Acc = accuracy, Sens = sensitivity, Spec = specificity.
(DOCX)

S1 Data (XLSX)
