# Supplementary figures and images for: Prediction of lymphoma response to CAR T cells by deep learning-based image analysis
Source: PLoS One. 2023 Jul 21;18(7):e0282573. doi: 10.1371/journal.pone.0282573 (PMC10361488; doi:10.1371/journal.pone.0282573)

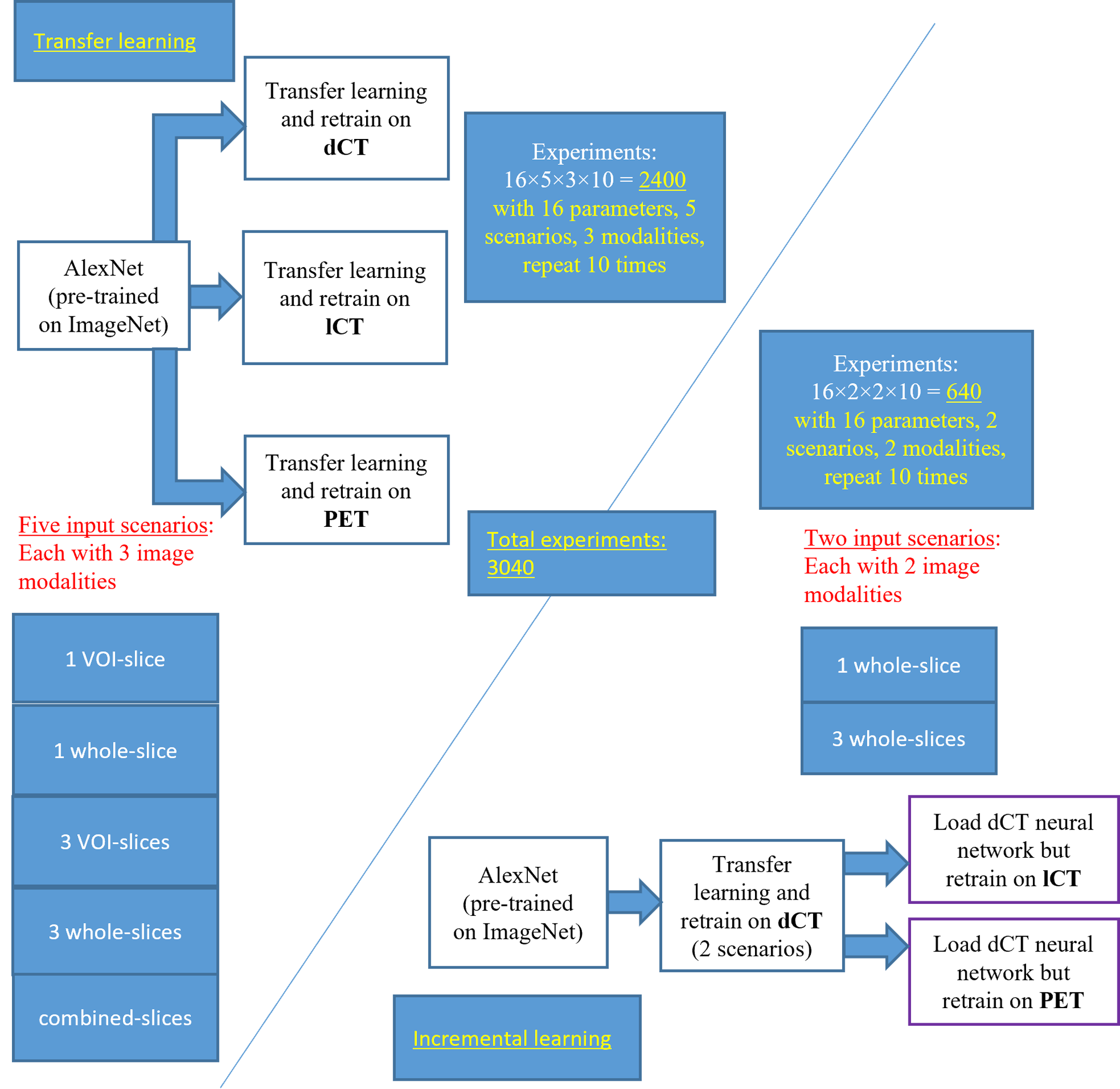

Supplement: S1 Fig — (TIF) [file pone.0282573.s001.tif]

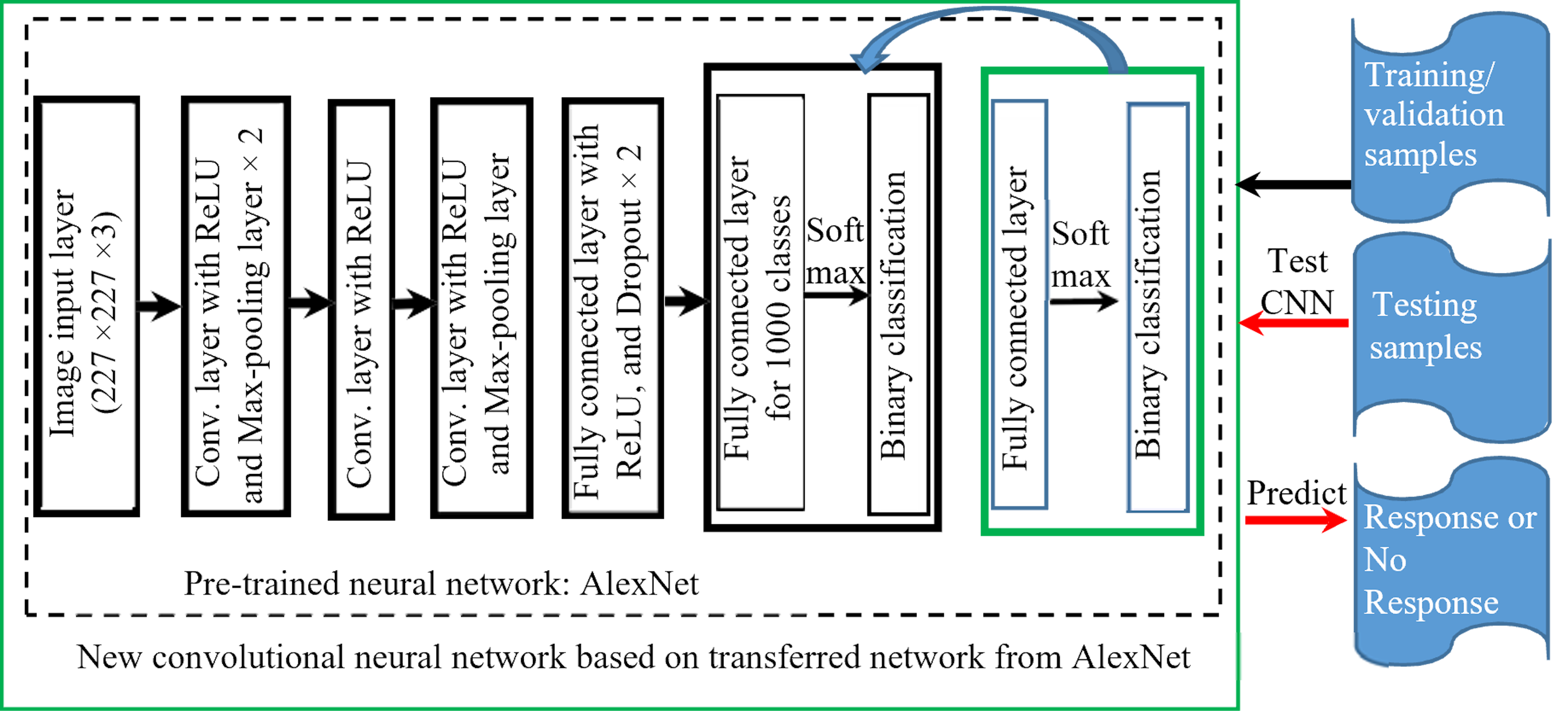

Supplement: S2 Fig — CNN = convolutional neural network, ReLU = rectified linear unit, Conv. = convolutional. (TIF) [file pone.0282573.s002.tif]

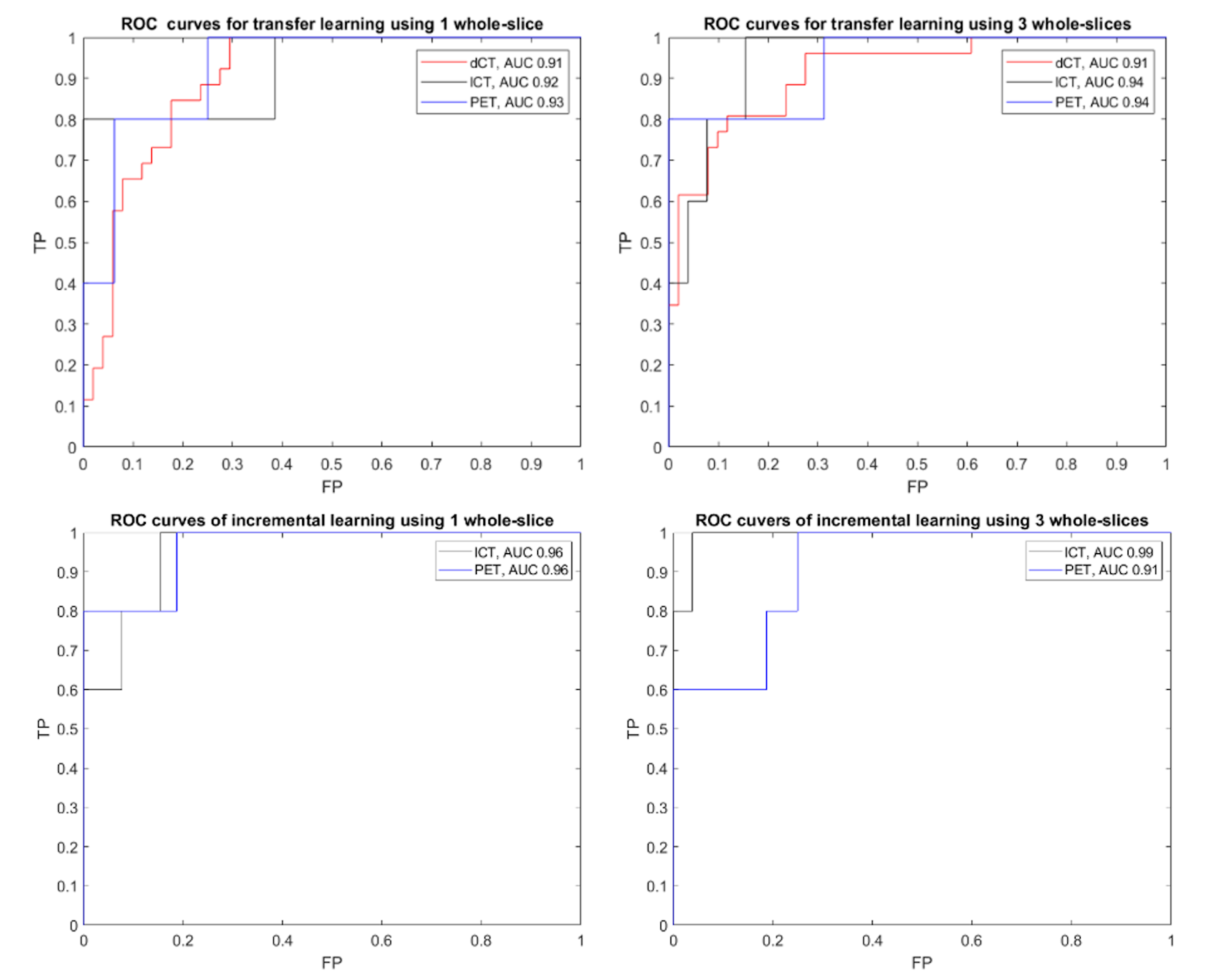

Supplement: S3 Fig — TP = true positive fraction, FP = false positive fraction, AUC = area under the curve, dCT = diagnostic computed tomography, lCT = low-dose computed tomography, PET = positron emission tomography. (TIF) [file pone.0282573.s003.tif]

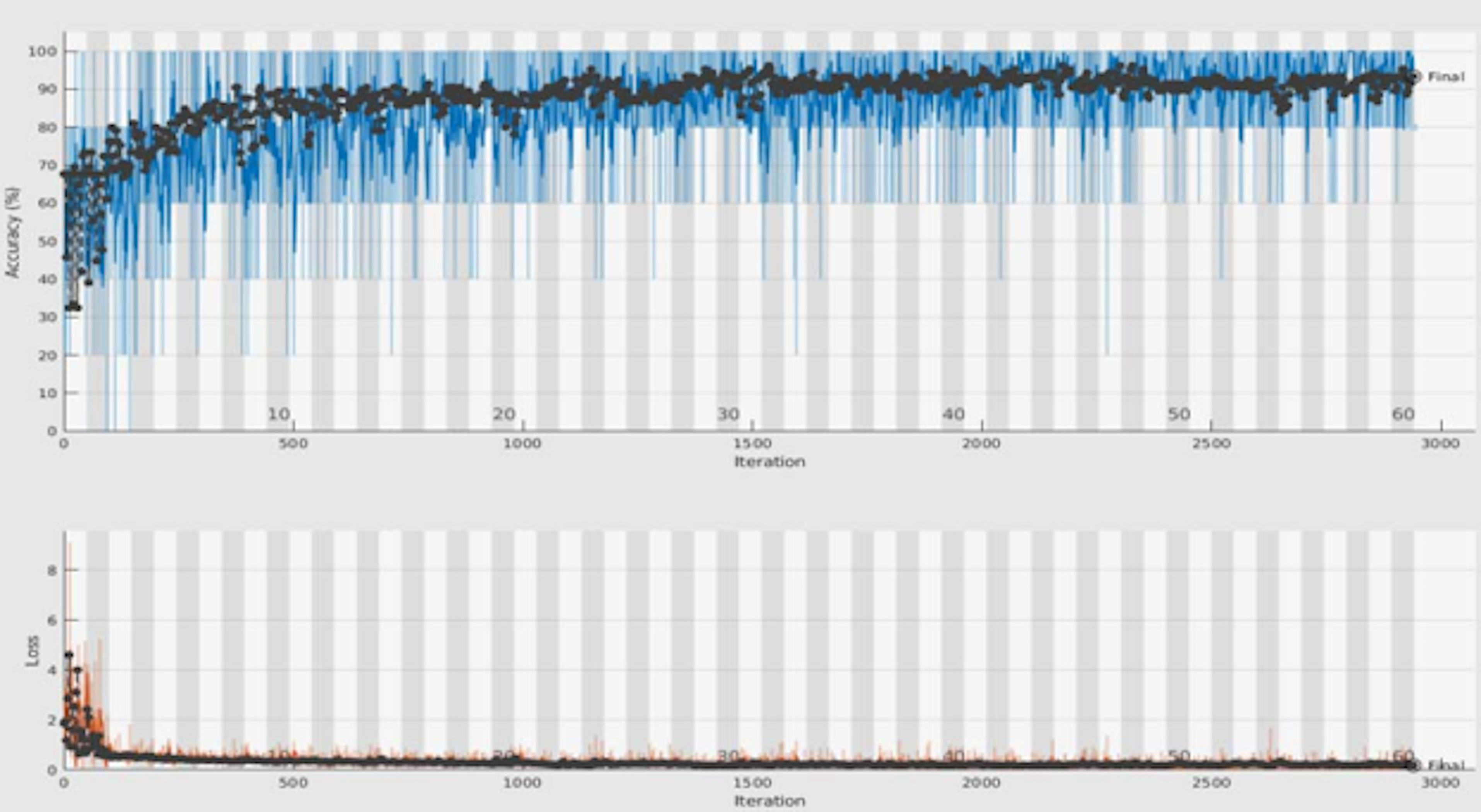

Supplement: S4 Fig — (TIF) [file pone.0282573.s004.tif]
