# Supplementary material for: Prediction of lymphoma response to CAR T cells by deep learning-based image analysis
Source: PLoS One. 2023 Jul 21;18(7):e0282573. doi: 10.1371/journal.pone.0282573 (PMC10361488; doi:10.1371/journal.pone.0282573)
Supplement: S1 Table — Patients categorized as (1) full responders (F-R) (i.e., where all lesions responded), (2) full non-responders (F-NR) (i.e., where no lesions responded), and (3) partial responders (P-R) (i.e., where only some lesions responded). dCT = diagnostic computed tomography, lCT = low-dose computed tomography, PET = positron emission tomography. (DOCX) [file pone.0282573.s005.docx]

| **S1 Table. Summary of response categories of lymphoma patients who received CAR T-cell therapy. Patients categorized as (1) full responders (F-R) (i.e., where all lesions responded), (2) full non-responders (F-NR) (i.e., where no lesions responded), and (3) partial responders (P-R) (i.e., where only some lesions responded). dCT = diagnostic computed tomography, lCT = low-dose computed tomography, PET = positron emission tomography.** | | | | | | |
| --- | --- | --- | --- | --- | --- | --- |
|  | **dCT** | | **lCT** | | **PET** | |
| **Patient response category groups** | **Number of lesions** | **Number of patients** | **Number of lesions** | **Number**  **of patients** | **Number of lesions** | **Number**  **of patients** |
| **Full responders**  **(F-R)** | 209 | 13 | 142 | 12 | 93 | 9 |
| **Full non-responders**  **(F-NR)** | 62 | 8 | 27 | 4 | 16 | 2 |
| **Partial responders (P-R) – responding**  **lesions** | 76 | 7 | 22 | 6 | 21 | 6 |
| **Partial responders (P-NR) – non-**  **responding lesions** | 55 |  | 23 |  | 24 |  |
| **Total** | 402 | 28 | 214 | 22 | 154 | 17 |
