# Supplementary material for: Prediction of lymphoma response to CAR T cells by deep learning-based image analysis
Source: PLoS One. 2023 Jul 21;18(7):e0282573. doi: 10.1371/journal.pone.0282573 (PMC10361488; doi:10.1371/journal.pone.0282573)
Supplement: S2 Table — dCT = diagnostic computed tomography, lCT = low-dose computed tomography, PET = positron emission tomography, VOI = volume of interest. (DOCX) [file pone.0282573.s006.docx]

| **S2 Table. Experiments with transfer learning for lesion-level treatment response prediction. dCT = diagnostic computed tomography, lCT** **= low-dose computed tomography, PET = positron emission tomography, VOI = volume of interest.** | | | | |
| --- | --- | --- | --- | --- |
| **Imaging**  **modalities** | | **Experimental hyperparameters and input scenarios** | **Data sets** | **Methods** |
| **dCT** | | Batch size [5, 10, 20, 30],  epochs [40, 80, 100, 200]  1 VOI-slice, 3 VOI-slices, 1 whole-slice, 3 whole-slices, and combined-slices | 6:2:2 ratio of  training: validation: testing among 383 samples | Transfer learning with pre- trained AlexNet |
| **PET/CT** | **lCT** |  | 6:2:2 ratio of  training: validation: testing among 158 samples | Transfer learning with pre- trained AlexNet plus incremental learning from pre-  trained network for dCT |
|  | **PET** |  | 6:2:2 ratio of  training: validation: testing  among 108 samples | Transfer learning with pre- trained AlexNet plus incremental learning from pre-  trained network for dCT |
