# Supplementary material for: Prediction of lymphoma response to CAR T cells by deep learning-based image analysis
Source: PLoS One. 2023 Jul 21;18(7):e0282573. doi: 10.1371/journal.pone.0282573 (PMC10361488; doi:10.1371/journal.pone.0282573)
Supplement: S3 Table — Cells with statistically significant p values are highlighted. dCT = diagnostic computed tomography, lCT = low-dose computed tomography, PET = positron emission tomography, VOI = volume of interest, Acc = accuracy, Sens = sensitivity, Spec = specificity, AUC = area under the curve. (DOCX) [file pone.0282573.s007.docx]

| **S3 Table. P values of t-test comparisons of diagnostic performance between 5 input scenarios for lesion-level treatment response prediction in lymphoma. Cells with statistically significant p values are highlighted. dCT = diagnostic computed tomography, lCT = low-dose computed tomography, PET = positron emission tomography, VOI = volume of interest, Acc = accuracy, Sens = sensitivity, Spec = specificity, AUC = area under the curve.** | | | | | | | | | | | | |
| --- | --- | --- | --- | --- | --- | --- | --- | --- | --- | --- | --- | --- |
|  | **dCT** | | | | **lCT** | | | | **PET** | | | |
| **Testing** | **Acc** | **Sens** | **Spec** | **AUC** | **Acc** | **Sens** | **Spec** | **AUC** | **Acc** | **Sens** | **Spec** | **AUC** |
| **1 VOI-slice vs. 1 whole-slice** | <0.0001 | <0.0001 | 0.013 | <0.0001 | 0.003 | 0.001 | 0.1687 | <0.0001 | < 0.0001 | <0.0001 | 0.002 | <0.0001 |
| **1 VOI-slice vs. 3 VOI-slices** | 0.308 | 0.049 | 0.442 | 0.016 | 0.247 | 0.108 | 0.12 | 0.2354 | <0.0001 | <0.0001 | 0.036 | 0.653 |
| **1 VOI-slice vs. 3 whole-slices** | <0.0001 | <0.0001 | 0.017 | <0.0001 | 0.005 | <0.0001 | 0.149 | <0.0001 | < 0.0001 | <0.001 | 0.0015 | <0.0001 |
| **1 whole-slice vs. 3 VOI-slices** | <0.0001 | <0.0001 | 0.006 | <0.0001 | 0.0002 | 0.0003 | 0.002 | <0.0001 | <0.001 | <0.001 | 0.0006 | <0.0001 |
| **1 whole-slice vs. 3 whole-slices** | 0.446 | 0.192 | 0.880 | 0.528 | 0.602 | 0.493 | 0.946 | 0.657 | 0.287 | 0.055 | 0.638 | 0.464 |
| **3 VOI-slices vs. 3 whole-slices** | <0.0001 | <0.0001 | 0.007 | <0.0001 | 0.00021 | <0.0001 | 0.001 | <0.0001 | <0.0001 | <0.0001 | 0.0002 | <0.0001 |
| **1 whole-slice vs. combined-slices** | 0.41 | 0.30 | 0.76 | 0.98 | 0.36 | 0.35 | 0.46 | 0.07 | 0.91 | 0.31 | 0.51 | 0.70 |
