# Supplementary material for: Prediction of lymphoma response to CAR T cells by deep learning-based image analysis
Source: PLoS One. 2023 Jul 21;18(7):e0282573. doi: 10.1371/journal.pone.0282573 (PMC10361488; doi:10.1371/journal.pone.0282573)
Supplement: S4 Table — Cells with statistically significant p values are highlighted. dCT = diagnostic computed tomography, lCT = low-dose computed tomography, PET = positron emission tomography, Acc = accuracy, Sens = sensitivity, Spec = specificity, AUC = area under the curve. (DOCX) [file pone.0282573.s008.docx]

| **S4 Table. P values of t-test comparisons of diagnostic performance between 3 image modalities (for 1 whole-slice and 3 whole-slices input scenarios) for lesion-level treatment response prediction. Cells with statistically significant p values are highlighted. dCT = diagnostic computed tomography, lCT** **= low-dose computed tomography, PET = positron emission tomography, Acc = accuracy, Sens = sensitivity, Spec = specificity, AUC = area under the curve.** | | | | | | | | | |
| --- | --- | --- | --- | --- | --- | --- | --- | --- | --- |
| **1 whole-slice** | **Acc** | **Sens** | **Spec** | **AUC** | **3 whole-slices** | **Acc** | **Sens** | **Spec** | **AUC** |
| **dCT vs. lCT** | 0.002 | 0.027 | 0.89 | 0.71 | **dCT vs. lCT** | 0.01 | 0.01 | 0.85 | 0.18 |
| **dCT vs. PET** | 0.08 | 0.27 | 0.92 | 0.42 | **dCT vs. PET** | 0.04 | 0.02 | 0.44 | 0.05 |
| **lCT vs. PET** | 0.11 | 0.19 | 0.85 | 0.79 | **lCT vs. PET** | 1.00 | 0.97 | 0.43 | 0.61 |
