# Supplementary material for: Prediction of lymphoma response to CAR T cells by deep learning-based image analysis
Source: PLoS One. 2023 Jul 21;18(7):e0282573. doi: 10.1371/journal.pone.0282573 (PMC10361488; doi:10.1371/journal.pone.0282573)
Supplement: S5 Table — a. Diagnostic performance of lesion-level treatment response prediction in lymphoma from diagnostic computed tomography (dCT) images for 5 input scenarios (using 40 epochs and batch size 5). Mean and standard deviation values are displayed. VOI = volume of interest, AUC = area under the curve. b. Diagnostic performance of lesion-level treatment response prediction in lymphoma from low-dose computed tomography (lCT) images for 5 input scenarios (using 40 epochs and batch size 5). Mean and standard deviation values are displayed. VOI = volume of interest, AUC = area under the curve. c. Diagnostic performance of lesion-level treatment response prediction in lymphoma from positron emission tomography (PET) images for 5 input scenarios (using 40 epochs and batch size 5). Mean and standard deviation values are displayed. VOI = volume of interest, AUC = area under the curve. (ZIP) [file pone.0282573.s009.zip › S5a_Table.docx]

| **S5a Table. Diagnostic performance of lesion-level treatment response prediction in lymphoma from diagnostic computed tomography (dCT) images for 5 input scenarios (using 40 epochs and batch size 5). Mean and standard deviation values are displayed. VOI = volume of interest, AUC = area under the curve.** | | | | | |
| --- | --- | --- | --- | --- | --- |
| **Input scenario** | **Task** | **Accuracy** | **Sensitivity** | **Specificity** | **AUC** |
| **1 VOI-slice** | **Training** | 0.81 ± 0.04 | 0.79 ± 0.05 | 0.93 ± 0.05 | 0.83 ± 0.08 |
|  | **Validation** | 0.66 ± 0.04 | 0.69 ± 0.02 | 0.52 ± 0.16 | 0.58 ± 0.06 |
|  | **Testing** | 0.68 ± 0.05 | 0.70 ± 0.02 | 0.58 ± 0.16 | 0.59 ± 0.04 |
| **1 whole-slice** | **Training** | 0.90 ± 0.03 | 0.92 ± 0.05 | 0.87 ± 0.09 | 0.97 ± 0.01 |
|  | **Validation** | 0.81 ± 0.04 | 0.85 ± 0.04 | 0.76 ± 0.11 | 0.89 ± 0.03 |
|  | **Testing** | 0.82 ± 0.05 | 0.87 ± 0.07 | 0.77 ± 0.12 | 0.91 ± 0.03 |
| **3 VOI-slices** | **Training** | 0.77 ± 0.03 | 0.75 ± 0.03 | 0.89 ± 0.05 | 0.80 ± 0.04 |
|  | **Validation** | 0.65 ± 0.03 | 0.68 ± 0.02 | 0.46 ± 0.12 | 0.55 ± 0.06 |
|  | **Testing** | 0.65 ± 0.05 | 0.68 ± 0.02 | 0.52 ± 0.21 | 0.52 ± 0.07 |
| **3 whole-slices** | **Training** | 0.94 ± 0.02 | 0.96 ± 0.03 | 0.91 ± 0.06 | 0.98 ± 0.01 |
|  | **Validation** | 0.83 ± 0.04 | 0.90 ± 0.05 | 0.74 ± 0.09 | 0.90 ± 0.03 |
|  | **Testing** | 0.84 ± 0.05 | 0.90 ± 0.04 | 0.76 ± 0.12 | 0.90 ± 0.05 |
| **combined-slices** | **Training** | 0.95 ± 0.03 | 0.97 ± 0.03 | 0.93 ± 0.05 | 0.99 ± 0.01 |
|  | **Validation** | 0.83 ± 0.03 | 0.87 ± 0.04 | 0.78 ± 0.07 | 0.90 ± 0.04 |
|  | **Testing** | 0.84 ± 0.04 | 0.89 ± 0.03 | 0.76 ± 0.10 | 0.91 ± 0.03 |
