# Supplementary material for: Prediction of lymphoma response to CAR T cells by deep learning-based image analysis
Source: PLoS One. 2023 Jul 21;18(7):e0282573. doi: 10.1371/journal.pone.0282573 (PMC10361488; doi:10.1371/journal.pone.0282573)
Supplement: S5 Table — a. Diagnostic performance of lesion-level treatment response prediction in lymphoma from diagnostic computed tomography (dCT) images for 5 input scenarios (using 40 epochs and batch size 5). Mean and standard deviation values are displayed. VOI = volume of interest, AUC = area under the curve. b. Diagnostic performance of lesion-level treatment response prediction in lymphoma from low-dose computed tomography (lCT) images for 5 input scenarios (using 40 epochs and batch size 5). Mean and standard deviation values are displayed. VOI = volume of interest, AUC = area under the curve. c. Diagnostic performance of lesion-level treatment response prediction in lymphoma from positron emission tomography (PET) images for 5 input scenarios (using 40 epochs and batch size 5). Mean and standard deviation values are displayed. VOI = volume of interest, AUC = area under the curve. (ZIP) [file pone.0282573.s009.zip › S5c_Table.docx]

| **S5c Table. Diagnostic performance of lesion-level treatment response prediction in lymphoma from positron emission tomography (PET) images for 5 input scenarios (using 40 epochs and batch size 5). Mean and standard deviation values are displayed. VOI = volume of interest, AUC = area under the curve.** | | | | | |
| --- | --- | --- | --- | --- | --- |
| **Input scenario** | **Task** | **Accuracy** | **Sensitivity** | **Specificity** | **AUC** |
| **1 VOI-slice** | **Training** | 0.81 ± 0.04 | 0.79 ± 0.05 | 0.93 ± 0.05 | 0.91 ± 0.01 |
|  | **Validation** | 0.66 ± 0.04 | 0.69 ± 0.02 | 0.52 ± 0.16 | 0.59 ± 0.13 |
|  | **Testing** | 0.68 ± 0.05 | 0.70 ± 0.02 | 0.58 ± 0.16 | 0.51 ± 0.14 |
| **1 whole-slice** | **Training** | 0.97 ± 0.03 | 0.97 ± 0.03 | 0.95 ± 0.07 | 1.00 ± 0.00 |
|  | **Validation** | 0.89 ± 0.03 | 0.94 ± 0.04 | 0.75 ± 0.11 | 0.93 ± 0.06 |
|  | **Testing** | 0.87 ± 0.06 | 0.90 ± 0.06 | 0.77 ± 0.19 | 0.93 ± 0.07 |
| **3 VOI-slices** | **Training** | 0.77 ± 0.01 | 0.75 ± 0.01 | 0.92 ± 0.08 | 0.80 ± 0.04 |
|  | **Validation** | 0.66 ± 0.03 | 0.68 ± 0.02 | 0.56 ± 0.21 | 0.53 ± 0.07 |
|  | **Testing** | 0.65 ± 0.02 | 0.68 ± 0.01 | 0.48 ± 0.09 | 0.53 ± 0.07 |
| **3 whole-slices** | **Training** | 0.95 ± 0.02 | 0.98 ± 0.02 | 0.90 ± 0.07 | 0.99 ± 0.01 |
|  | **Validation** | 0.89 ± 0.06 | 0.93 ± 0.04 | 0.78 ± 0.17 | 0.92 ± 0.05 |
|  | **Testing** | 0.90 ± 0.07 | 0.95 ± 0.05 | 0.81 ± 0.19 | 0.95 ± 0.06 |
| **combined-slices** | **Training** | 0.98 ± 0.02 | 0.99 ± 0.02 | 0.97 ± 0.05 | 1.00 ± 0.01 |
|  | **Validation** | 0.81 ± 0.09 | 0.90 ± 0.06 | 0.59 ± 0.18 | 0.86 ± 0.10 |
|  | **Testing** | 0.87 ± 0.07 | 0.93 ± 0.07 | 0.73 ± 0.16 | 0.92 ± 0.08 |

| **S6 Table. Diagnostic performance of lesion-level treatment response prediction in lymphoma using incremental learning vs. transfer learning (for 2 input scenarios) on low-dose computed tomography (lCT) and positron emission tomography (PET) image modalities. Mean and standard deviation values are displayed. Acc = accuracy, Sens = sensitivity, Spec = specificity, AUC = area under the curve.** | | | | | | | | |
| --- | --- | --- | --- | --- | --- | --- | --- | --- |
|  | **1 whole-slice** | | | | **3 whole-slices** | | | |
|  | **Acc** | **Sens** | **Spec** | **AUC** | **Acc** | **Sens** | **Spec** | **AUC** |
| **Incremental learning** | 0.93 | 0.96 | 0.82 | 0.96 | 0.98 | 0.99 | 0.93 | 0.99 |
| **on lCT** | ±0.03 | ±0.03 | ±0.16 | ±0.04 | ±0.02 | ±0.02 | ±0.09 | ±0.02 |
| **Transfer learning** | 0.91 | 0.94 | 0.75 | 0.92 | 0.90 | 0.95 | 0.74 | 0.94 |
| **on lCT** | ±0.06 | ±0.06 | ±0.32 | ±0.08 | ±0.05 | ±0.04 | ±0.20 | ±0.07 |
| **p values** | 0.46 | 0.37 | 0.52 | 0.19 | <0.001 | 0.02 | 0.02 | 0.05 |
|  | | | | | | | | |
| **Incremental learning** | 0.86 | 0.95 | 0.70 | 0.96 | 0.86 | 0.91 | 0.72 | 0.92 |
| **on PET** | ±0.07 | ±0.04 | ±0.18 | ±0.04 | ±0.07 | ±0.04 | ±0.16 | ±0.06 |
| **Transfer learning** | 0.87 | 0.90 | 0.77 | 0.93 | 0.90 | 0.95 | 0.81 | 0.95 |
| **on PET** | ±0.06 | ±0.06 | ±0.19 | ±0.07 | ±0.07 | ±0.05 | ±0.19 | ±0.06 |
| **p values** | 0.88 | 0.04 | 0.36 | 0.30 | 0.20 | 0.05 | 0.26 | 0.18 |

| **S7 Table. Diagnostic performance of lesion-level treatment response prediction in lymphoma using transfer learning on 1 whole-slice input scenario from diagnostic computed tomography (dCT) based on different hyperparameters of batch size (B) and number of epochs (E). Mean and standard deviation values are displayed. Acc = accuracy, Sens = sensitivity, Spec = specificity, AUC = area under the curve.** | | | | | | | | | | | | | | | | | | | |
| --- | --- | --- | --- | --- | --- | --- | --- | --- | --- | --- | --- | --- | --- | --- | --- | --- | --- | --- | --- |
| **Hyp**  **erpa**  **ram**  **eters** | **Acc** | **Sens** | **Spec** | **AUC** | **Hyperparameters** | **Acc** | **Sens** | **Spec** | **AUC** | **Hyperparameters** | **Acc** | **Sens** | **Spec** | **AUC** | **Hyperparameters** | **Acc** | **Sens** | **Spec** | **AUC** |
| **B5** | 0.82 | 0.87 | 0.77 | 0.91 | **B10** | 0.82 | 0.89 | 0.71 | 0.91 | **B20** | 0.81 | 0.86 | 0.71 | 0.89 | **B30** | 0.79 | 0.86 | 0.68 | 0.85 |
| **E40** | ±0.05 | ±0.07 | ±0.12 | ±0.03 | **E40** | ±0.04 | ±0.05 | ±0.07 | ±0.02 | **E40** | ±0.03 | ±0.05 | ±0.05 | ±0.03 | **E40** | ±0.06 | ±0.04 | ±0.11 | ±0.05 |
| **B5** | 0.82 | 0.84 | 0.77 | 0.89 | **B10** | 0.85 | 0.89 | 0.79 | 0.90 | **B20** | 0.85 | 0.89 | 0.77 | 0.89 | **B30** | 0.82 | 0.87 | 0.73 | 0.87 |
| **E80** | ±0.03 | ±0.03 | ±0.09 | ±0.05 | **E80** | ±0.03 | ±0.04 | ±0.05 | ±0.03 | **E80** | ±0.04 | ±0.05 | ±0.07 | ±0.04 | **E80** | ±0.06 | ±0.04 | ±0.09 | ±0.05 |
| **B5** | 0.85 | 0.88 | 0.78 | 0.93 | **B10** | 0.85 | 0.88 | 0.82 | 0.91 | **B20** | 0.84 | 0.88 | 0.76 | 0.89 | **B30** | 0.82 | 0.87 | 0.72 | 0.87 |
| **E100** | ±0.03 | ±0.04 | ±0.06 | ±0.02 | **E100** | ±0.03 | ±0.04 | ±0.09 | ±0.04 | **E100** | ±0.02 | ±0.04 | ±0.03 | ±0.03 | **E100** | ±0.03 | ±0.04 | ±0.05 | ±0.04 |
| **B5** | 0.87 | 0.90 | 0.83 | 0.93 | **B10** | 0.87 | 0.90 | 0.83 | 0.89 | **B20** | 0.82 | 0.87 | 0.74 | 0.90 | **B30** | 0.83 | 0.86 | 0.78 | 0.89 |
| **E200** | ±0.04 | ±0.05 | ±0.08 | ±0.02 | **E200** | ±0.05 | ±0.04 | ±0.08 | ±0.05 | **E200** | ±0.02 | ±0.03 | ±0.05 | ±0.04 | **E200** | ±0.03 | ±0.03 | ±0.08 | ±0.03 |

| **S8 Table. P values of t-test comparisons of diagnostic performance between selected hyperparameter combinations of transfer learning (from Table S7) for lesion-level treatment response prediction (using 1 whole-slice input scenario from diagnostic computed tomography (dCT)). Cells with statistically significant p values are highlighted. B = batch size, E = number of epochs, Acc = accuracy, Sens = sensitivity, Spec = specificity, AUC = area under the curve.** | | | | |
| --- | --- | --- | --- | --- |
| **Hyperparameter comparisons** | **Acc** | **Sens** | **Spec** | **AUC** |
| **B5 E40 vs. B5 E200** | 0.03 | 0.28 | 0.19 | 0.21 |
| **B10 E40 vs. B10 E200** | 0.03 | 0.40 | 0.02 | 0.19 |
| **B20 E40 vs. B20 E200** | 0.16 | 0.20 | 0.29 | 0.65 |
| **B30 E40 vs. B30 E200** | 0.12 | 0.88 | 0.08 | 0.12 |
| **B5 E40 vs. B30 E40** | 0.24 | 0.87 | 0.09 | 0.01 |
| **B5 E80 vs. B30 E80** | 0.003 | 0.009 | 0.0003 | 0.26 |
| **B5 E100 vs. B30 E100** | 0.03 | 0.51 | 0.02 | 0.001 |
| **B5 E200 vs. B30 E200** | 0.02 | 0.05 | 0.12 | 0.002 |

| **S9 Table. Diagnostic performance of patient-level treatment response prediction in lymphoma using rule-based reasoning approach (from lesion-level response predictions using 3 whole-slices input scenario, 3 image modalities, and transfer learning) compared to International Prognostic Index risk factors for diffuse large B-cell lymphoma (DLBCL) patients. Note that results are shown for entire subject cohort (All) and for DLBCL subject cohort. dCT = diagnostic computed tomography, lCT = low-dose computed tomography, PET = positron emission tomography, IPI = International Prognostic Index, Acc = accuracy, Sens = sensitivity, Spec = specificity.** | | | | | | | | | | |
| --- | --- | --- | --- | --- | --- | --- | --- | --- | --- | --- |
|  |  | **Patient response with**  **"All" Rule** | | | **Patient response with**  **"Majority" Rule** | | | | | |
| **Subject cohort** | **Modality** | **All lesions**  **Responded** | | | **At least "60%" lesions responded** | | | **At least "70%"**  **lesions responded** | | |
|  |  | **Acc** | **Sens** | **Spec** | **Acc** | **Sens** | **Spec** | **Acc** | **Sens** | **Spec** |
| **All** | **dCT** | 0.64 | 0.75 | 0.60 | 0.71 | 0.67 | 0.80 | 0.75 | 0.82 | 0.71 |
|  | **lCT** | 0.43 | 0.40 | 0.46 | 0.61 | 0.57 | 0.67 | 0.57 | 0.54 | 0.60 |
|  | **PET** | 0.35 | 0.29 | 0.40 | 0.47 | 0.45 | 0.50 | 0.41 | 0.40 | 0.43 |
| **DLBCL** | **dCT** | 0.69 | 0.75 | 0.67 | 0.69 | 0.60 | 0.83 | 0.81 | 1.00 | 0.75 |
|  | **lCT** | 0.50 | 0.40 | 0.63 | 0.56 | 0.45 | 0.71 | 0.56 | 0.45 | 0.71 |
|  | **PET** | 0.38 | 0.29 | 0.50 | 0.38 | 0.33 | 0.50 | 0.38 | 0.33 | 0.50 |
|  | **IPI ≤ 1** | Acc = 0.54; Sens = 0.38; Spec = 0.61 | | | | | | | | |
|  | **IPI ≤ 2** | Acc = 0.42; Sens = 0.37; Spec = 0.57 | | | | | | | | |
|  | **IPI ≤ 3** | Acc = 0.27; Sens = 0.30; Spec = 0.00 | | | | | | | | |
