# Supplementary material for: Prediction of lymphoma response to CAR T cells by deep learning-based image analysis
Source: PLoS One. 2023 Jul 21;18(7):e0282573. doi: 10.1371/journal.pone.0282573 (PMC10361488; doi:10.1371/journal.pone.0282573)
Supplement: S7 Table — Mean and standard deviation values are displayed. Acc = accuracy, Sens = sensitivity, Spec = specificity, AUC = area under the curve. (DOCX) [file pone.0282573.s011.docx]

| **S7 Table. Diagnostic performance of lesion-level treatment response prediction in lymphoma using transfer learning on 1 whole-slice input scenario from diagnostic computed tomography (dCT) based on different hyperparameters of batch size (B) and number of epochs (E). Mean and standard deviation values are displayed. Acc = accuracy, Sens = sensitivity, Spec = specificity, AUC = area under the curve.** | | | | | | | | | | | | | | | | | | | |
| --- | --- | --- | --- | --- | --- | --- | --- | --- | --- | --- | --- | --- | --- | --- | --- | --- | --- | --- | --- |
| **Hyp**  **erpa**  **ram**  **eters** | **Acc** | **Sens** | **Spec** | **AUC** | **Hyperparameters** | **Acc** | **Sens** | **Spec** | **AUC** | **Hyperparameters** | **Acc** | **Sens** | **Spec** | **AUC** | **Hyperparameters** | **Acc** | **Sens** | **Spec** | **AUC** |
| **B5** | 0.82 | 0.87 | 0.77 | 0.91 | **B10** | 0.82 | 0.89 | 0.71 | 0.91 | **B20** | 0.81 | 0.86 | 0.71 | 0.89 | **B30** | 0.79 | 0.86 | 0.68 | 0.85 |
| **E40** | ±0.05 | ±0.07 | ±0.12 | ±0.03 | **E40** | ±0.04 | ±0.05 | ±0.07 | ±0.02 | **E40** | ±0.03 | ±0.05 | ±0.05 | ±0.03 | **E40** | ±0.06 | ±0.04 | ±0.11 | ±0.05 |
| **B5** | 0.82 | 0.84 | 0.77 | 0.89 | **B10** | 0.85 | 0.89 | 0.79 | 0.90 | **B20** | 0.85 | 0.89 | 0.77 | 0.89 | **B30** | 0.82 | 0.87 | 0.73 | 0.87 |
| **E80** | ±0.03 | ±0.03 | ±0.09 | ±0.05 | **E80** | ±0.03 | ±0.04 | ±0.05 | ±0.03 | **E80** | ±0.04 | ±0.05 | ±0.07 | ±0.04 | **E80** | ±0.06 | ±0.04 | ±0.09 | ±0.05 |
| **B5** | 0.85 | 0.88 | 0.78 | 0.93 | **B10** | 0.85 | 0.88 | 0.82 | 0.91 | **B20** | 0.84 | 0.88 | 0.76 | 0.89 | **B30** | 0.82 | 0.87 | 0.72 | 0.87 |
| **E100** | ±0.03 | ±0.04 | ±0.06 | ±0.02 | **E100** | ±0.03 | ±0.04 | ±0.09 | ±0.04 | **E100** | ±0.02 | ±0.04 | ±0.03 | ±0.03 | **E100** | ±0.03 | ±0.04 | ±0.05 | ±0.04 |
| **B5** | 0.87 | 0.90 | 0.83 | 0.93 | **B10** | 0.87 | 0.90 | 0.83 | 0.89 | **B20** | 0.82 | 0.87 | 0.74 | 0.90 | **B30** | 0.83 | 0.86 | 0.78 | 0.89 |
| **E200** | ±0.04 | ±0.05 | ±0.08 | ±0.02 | **E200** | ±0.05 | ±0.04 | ±0.08 | ±0.05 | **E200** | ±0.02 | ±0.03 | ±0.05 | ±0.04 | **E200** | ±0.03 | ±0.03 | ±0.08 | ±0.03 |
