# Supplementary material for: Prediction of lymphoma response to CAR T cells by deep learning-based image analysis
Source: PLoS One. 2023 Jul 21;18(7):e0282573. doi: 10.1371/journal.pone.0282573 (PMC10361488; doi:10.1371/journal.pone.0282573)
Supplement: S8 Table — Cells with statistically significant p values are highlighted. B = batch size, E = number of epochs, Acc = accuracy, Sens = sensitivity, Spec = specificity, AUC = area under the curve. (DOCX) [file pone.0282573.s012.docx]

| **S8 Table. P values of t-test comparisons of diagnostic performance between selected hyperparameter combinations of transfer learning (from Table S7) for lesion-level treatment response prediction (using 1 whole-slice input scenario from diagnostic computed tomography (dCT)). Cells with statistically significant p values are highlighted. B = batch size, E = number of epochs, Acc = accuracy, Sens = sensitivity, Spec = specificity, AUC = area under the curve.** | | | | |
| --- | --- | --- | --- | --- |
| **Hyperparameter comparisons** | **Acc** | **Sens** | **Spec** | **AUC** |
| **B5 E40 vs. B5 E200** | 0.03 | 0.28 | 0.19 | 0.21 |
| **B10 E40 vs. B10 E200** | 0.03 | 0.40 | 0.02 | 0.19 |
| **B20 E40 vs. B20 E200** | 0.16 | 0.20 | 0.29 | 0.65 |
| **B30 E40 vs. B30 E200** | 0.12 | 0.88 | 0.08 | 0.12 |
| **B5 E40 vs. B30 E40** | 0.24 | 0.87 | 0.09 | 0.01 |
| **B5 E80 vs. B30 E80** | 0.003 | 0.009 | 0.0003 | 0.26 |
| **B5 E100 vs. B30 E100** | 0.03 | 0.51 | 0.02 | 0.001 |
| **B5 E200 vs. B30 E200** | 0.02 | 0.05 | 0.12 | 0.002 |
