# Supplementary material for: Prediction of lymphoma response to CAR T cells by deep learning-based image analysis
Source: PLoS One. 2023 Jul 21;18(7):e0282573. doi: 10.1371/journal.pone.0282573 (PMC10361488; doi:10.1371/journal.pone.0282573)
Supplement: S9 Table — Note that results are shown for entire subject cohort (All) and for DLBCL subject cohort. dCT = diagnostic computed tomography, lCT = low-dose computed tomography, PET = positron emission tomography, IPI = International Prognostic Index, Acc = accuracy, Sens = sensitivity, Spec = specificity. (DOCX) [file pone.0282573.s013.docx]

| **S9 Table. Diagnostic performance of patient-level treatment response prediction in lymphoma using rule-based reasoning approach (from lesion-level response predictions using 3 whole-slices input scenario, 3 image modalities, and transfer learning) compared to International Prognostic Index risk factors for diffuse large B-cell lymphoma (DLBCL) patients. Note that results are shown for entire subject cohort (All) and for DLBCL subject cohort. dCT = diagnostic computed tomography, lCT = low-dose computed tomography, PET = positron emission tomography, IPI = International Prognostic Index, Acc = accuracy, Sens = sensitivity, Spec = specificity.** | | | | | | | | | | |
| --- | --- | --- | --- | --- | --- | --- | --- | --- | --- | --- |
|  |  | **Patient response with**  **"All" Rule** | | | **Patient response with**  **"Majority" Rule** | | | | | |
| **Subject cohort** | **Modality** | **All lesions**  **Responded** | | | **At least "60%" lesions responded** | | | **At least "70%"**  **lesions responded** | | |
|  |  | **Acc** | **Sens** | **Spec** | **Acc** | **Sens** | **Spec** | **Acc** | **Sens** | **Spec** |
| **All** | **dCT** | 0.64 | 0.75 | 0.60 | 0.71 | 0.67 | 0.80 | 0.75 | 0.82 | 0.71 |
|  | **lCT** | 0.43 | 0.40 | 0.46 | 0.61 | 0.57 | 0.67 | 0.57 | 0.54 | 0.60 |
|  | **PET** | 0.35 | 0.29 | 0.40 | 0.47 | 0.45 | 0.50 | 0.41 | 0.40 | 0.43 |
| **DLBCL** | **dCT** | 0.69 | 0.75 | 0.67 | 0.69 | 0.60 | 0.83 | 0.81 | 1.00 | 0.75 |
|  | **lCT** | 0.50 | 0.40 | 0.63 | 0.56 | 0.45 | 0.71 | 0.56 | 0.45 | 0.71 |
|  | **PET** | 0.38 | 0.29 | 0.50 | 0.38 | 0.33 | 0.50 | 0.38 | 0.33 | 0.50 |
|  | **IPI ≤ 1** | Acc = 0.54; Sens = 0.38; Spec = 0.61 | | | | | | | | |
|  | **IPI ≤ 2** | Acc = 0.42; Sens = 0.37; Spec = 0.57 | | | | | | | | |
|  | **IPI ≤ 3** | Acc = 0.27; Sens = 0.30; Spec = 0.00 | | | | | | | | |
